# Accelerometer‐measured weekend catch‐up sleep and incident dementia: a prospective cohort study

**DOI:** 10.1002/alz70860_102867

**Published:** 2025-12-23

**Authors:** Ting Shen, Hui Chen, Mengjia Zhao, Lusha Tong, Xiao Tan, Geng Zong, Tianyi Huang, Changzheng Yuan

**Affiliations:** ^1^ Zhejiang University School of Medicine, Hangzhou, Zhejiang, China; ^2^ Zhejiang University, Hangzhou, Zhejiang, China; ^3^ Shanghai Institute of Nutrition and Health, Chinese Academy of Sciences, Shanghai, China; ^4^ National Institute on Aging, Baltimore, MD, USA; ^5^ Harvard T. H. Chan School of Public Health, Boston, MA, USA

## Abstract

**Background:**

Previous studies have suggested the key role of healthy sleep duration in the prevention of dementia. However, the role of weekend catch‐up sleep, defined as extending sleep over the weekend to compensate for weekday inadequate sleep, was unclear.

**Method:**

From the UK Biobank, we included 83,776 dementia‐free adults aged 50 years or older (mean age: 63.3 years, standard deviation: 6.7 years; female: 56.5%) at the time of seven‐day wrist accelerometer assessment (2013‐2015). We followed them up until December 2022. Daily sleep duration was derived with a machine learning approach from the seven‐day wrist‐worn accelerometer data. Weekend catch‐up sleep was defined as the weekend‐weekday difference in average sleep duration. Incident all‐cause dementia was identified from linked healthcare data. Cox proportional hazard regression models were used to calculate hazard ratios (HRs) and 95% confidence intervals (CIs).

**Result:**

During a median follow‐up of 8.0 years with 667,928 person‐years, 713 participants developed all‐cause dementia. We observed a significant lower risk of dementia among individuals with a moderate level of weekend catch‐up sleep (1‐1.5 hours/day). In particular, the multi‐variable adjusted HRs across increasing categories of weekend catch‐up sleep (no or ≤0.5, >0.5‐1, >1‐1.5, >1.5‐2, and >2 hours) were 1 (reference), 0.91 (0.74‐1.11), 0.64 (0.49‐0.86), 0.84 (0.60‐1.16), and 0.83 (0.60‐1.16), respectively. The association between weekend catch‐up sleep and risk of dementia was stronger among participants with a weekday sleep duration <8 hours (HR: 0.49, 0.29‐0.81 for >1‐1.5 hours), but was non‐significant for participants with a weekday sleep duration ≥8 hours (HR: 0.72, 0.51‐1.01 for >1‐1.5 hours, *p*‐interaction = 0.039).

**Conclusion:**

Moderate accelerometer‐measured weekend catch‐up sleep was significantly associated with lower risk of all‐cause dementia. Future studies are needed to confirm the findings and determine the optimal duration for dementia prevention.

